# Supplementary material for: Myeloid Trem2 Dynamically Regulates the Induction and Resolution of Hepatic Ischemia-Reperfusion Injury Inflammation
Source: Int J Mol Sci. 2023 Mar 28;24(7):6348. doi: 10.3390/ijms24076348 (PMC10094065; doi:10.3390/ijms24076348)
Supplement: Supplementary file 1 [file ijms-24-06348-s001.zip › Supplementary Figure.pdf]

Supplementary Doc

Myeloid Trem2 dynamically regulates the induction and resolution of hepatic ischemia-reperfusion injury inflammation

Sheng Han[1,a,b,c], Xiangdong Li[1,a,b,c], Nan Xia[1,a,b,c], Yu Zhang[a,b,c], Wenjie Yu[a,b,c], Jie Li[a,b,c], Chenyu Jiao[a,b,c], Ziyi Wang[#,a,b,c], Liyong Pu[#,a,b,c]

a. Hepatobiliary Center, The First Affiliated Hospital of Nanjing Medical University, Nanjing, China
b. Key Laboratory of Liver Transplantation, Chinese Academy of Medical Sciences, Nanjing, China
c. NHC Key Laboratory of Living Donor Liver Transplantation (Nanjing Medical University), Nanjing,
China

Table of contents

Figure S1 2

Figure S2..........3

Figure S3 4

Figure S4 4

Figure S5 5

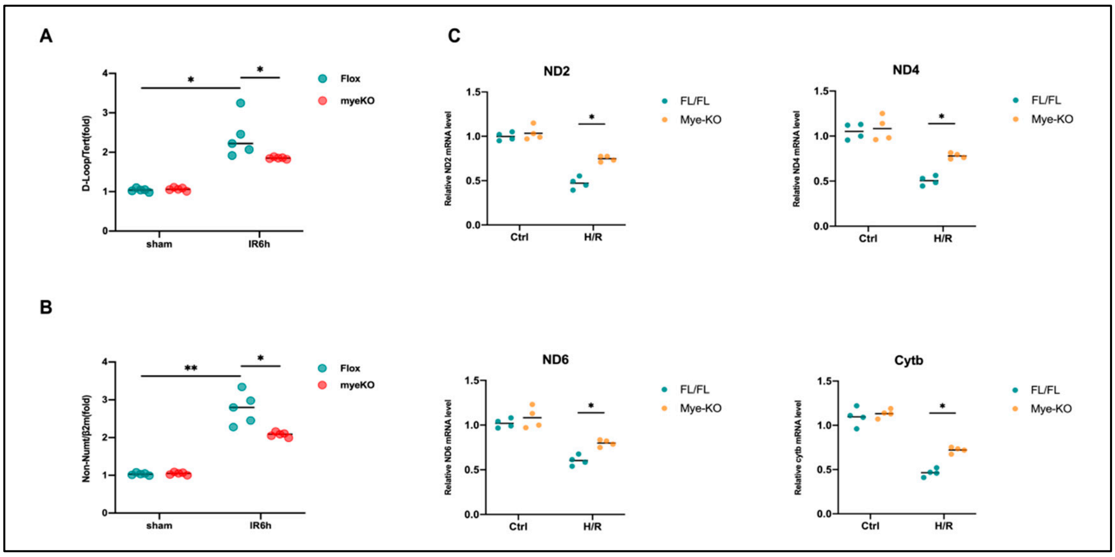

<u>Figure S1: Absence of Trem2 alleviates accumulation of mtDNA in BMDMs in respond to oxidative stress.</u>

(**A**) The mtDNA content detection was shown as the ratio of D-loop mtDNA to Tert nuclear (n) DNA or (**B**) mtDNA that is not inserted into nuclear DNA (non-NUMT) to B2m nDNA(n = 4-5 / group, *P < 0.05,** P < 0.01). (**C**) Detection of H/R-BMDM mitochondria-related genes ND2,ND4,ND6 and cytb by qRT-PCR;

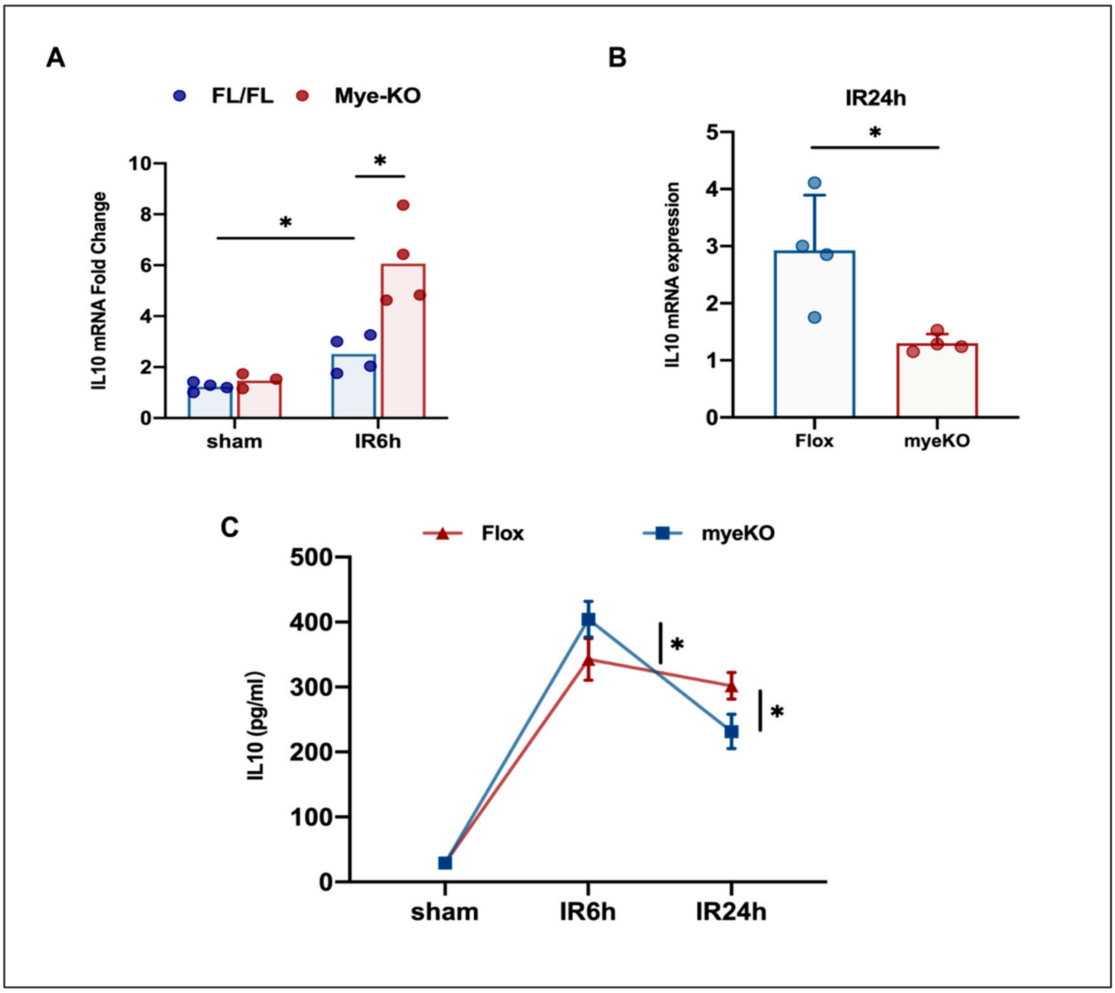

<u>Figure S2: myeloid TREM2 differentially regulates IL10 at different stages of IR.</u>

The effect of bone marrow TREM2 on IL10 expression at IR6h (**A**) and IR24h (**B**) was detected by qPCR; The IL10 content in the serum of mice at IR6h and IR24h was detected by ELISA (**C**) (* P < 0.05).

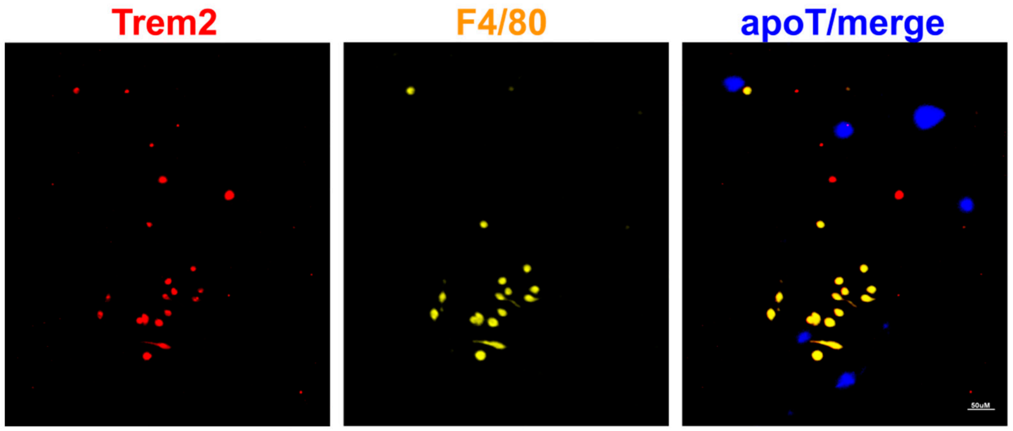

Figure S3: BMDM co-cultured with apoT at 4°C expressed TREM2.

The expression of TREM2 on BMDM co-cultured with apot at 37 ° C or 4 ° C was detected by ICC;

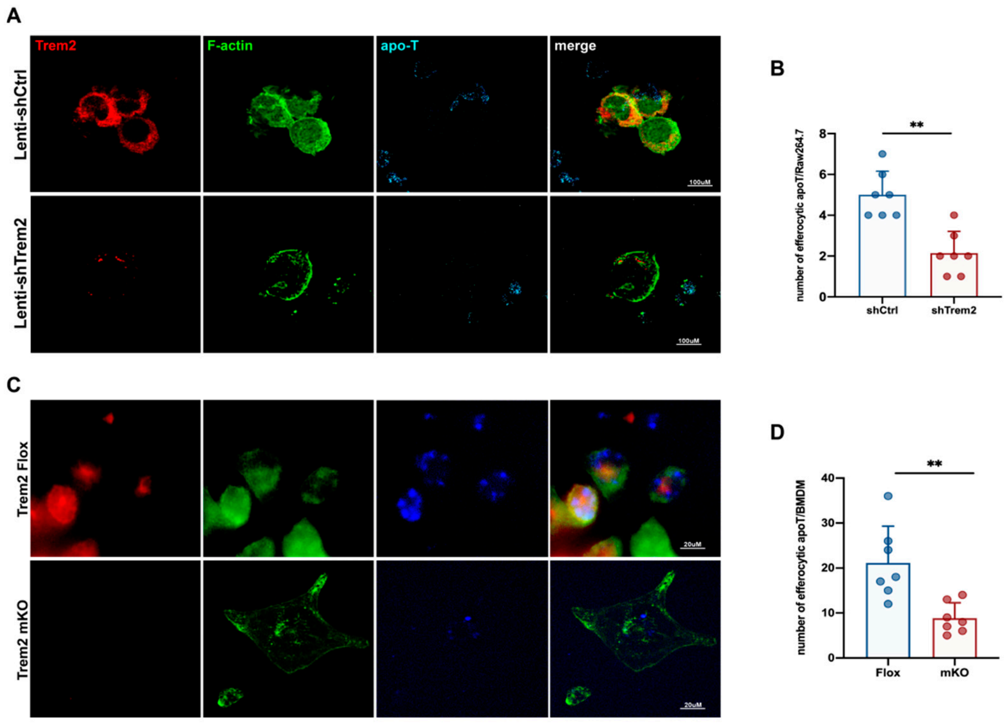

Figure S4: myeloid TREM2 drives actin remodeling of efferocytotic macrophages.

The efferocytosis program was started for RAW264.7 infected with shTrem2 or shCtr1 lentivirus, and then actin remodeling was judged by phalloidin staining (**A**) and the number of apoptotic cells phagocytized by each RAW264.7 was detected (**B**) (** $P < 0.01$); The efferocytosis program was initiated for BMDM of Trem2$^{FL/FL}$ or Trem2^{mKO}, respectively, and then actin remodeling was judged by phalloidin staining (**C**) and the number of apoptotic cells phagocytized by each bmdm was detected (**D**) (** $P < 0.01$)

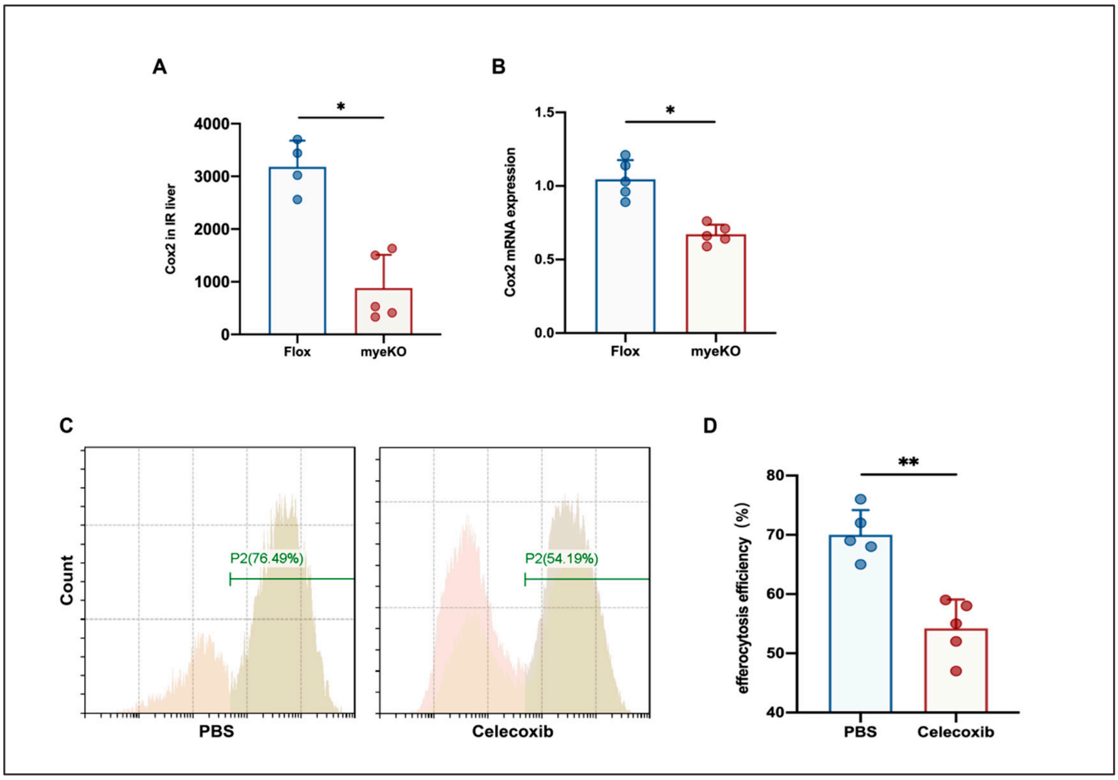

Figure S5. myeloid TREM2 determines the activation of macrophages COX2 that perform efferocytosis.

Abundance of COX2 (**A**) or mRNA transcription (**B**) the liver 24 h after IR was analyzed by mass spectrometry or qPCR (* $P < 0.05$); When BMDM of Trem2$^{FL/FL}$ or Trem2^{mKO} started efferocytosis program, the efficiency of efferocytosis was detected after adding celecoxib (**C** and **D**) (** $P < 0.01$).
